# Tom Hunt

**Published:** 1881-01

**Authors:** 


### TOM HUNT.

ON a frosty winter night Mrs. Hacker and her daughter sat in the little parlor behind the shop, taking tea. The brown teapot was kept on the back of the stove that it might not chill. So was the pan of sausages which tasted all the more crisp and savory in consequence. The door between the store and room stood open, that any chance customer might be seen at once by the tea drinkers, but the bell had not jingled since they took their seats.

"Trade is dreadful, Emma Jane," said Mrs. Hacker, dipping her bread into the sausage pan and transferring it to her plate by means of the long cooking fork.

"Trade is dreadful! I should just give it up if it got a little worse; but, dear me, I never had any luck in anything. There's Mr. Ninnever putting plate glass—whole panes—into his windows, and beginning to talk of hiring the second floor for ready-made suits; and my things hang on my hands, though I'm sure I make better selections than he knows how to. Another cup, Emma Jane—what a comfort tea is, to be sure."

"Then give me a cup, won't you, Mrs. Hacker?" said a voice behind them. "I want comfort, I'm sure. Here's grandma gone out and forgot to leave the key, and nothing for me to do but sit on the stairs and cool my heels."

"Thomas! la! how you scared me coming in so sudden!" screamed Emma Jane.

"Sit down, do," said Mrs. Hacker. "Take your seat here, Tom, and have your supper with us. Your grandma stopped to tell me she wouldn't be back until late, and the key is in the money-drawer."

"About all there is there, too," said Emma Jane, with a pout; "and I want a new winter bonnet."

"Look here, Mrs. Hacker," said the young man, slowly turning himself toward the old lady. "Look here, ma'am; here's some one ready and willing to buy that winter bonnet, and all the other bonnets Emma Jane will ever want. We've been engaged a year now, and at last I've got to be foreman in the factory. Why should we put it off any longer? Tell Emma Jane it's all nonsense. She won't listen to me."

"Well, I don't think long engagements are best," said Mrs. Hacker. "What I should say to Emma Jane would be, 'have him now.'"

"Well, I suppose I shall be bothered until I do say yes," replied Emma Jane, and then the anxious lover, pleading his cause earnestly, the wedding-day was actually set for Christmas eve, which was at that time about a month off.

This conversation, as well as the evening meal, being over, Mrs. Hacker discreetly retired to the shop and left the lovers alone. However, she did not stay away long. In a few minutes she came running in with her glasses on her nose, and an open letter in her hand.

"Read this, one of you," she said. "I've read it, but I can't believe I understand it. It seems as though I must be crazy. Here, you read it, Thomas; I have more confidence in you."

Then she put the letter into Thomas Hunt's hand and sat down at the table.

"I found it on the floor," said she. "The postmaster must have thrown it in at the slit. I don't know whether it's a hoax or not, but it's got a regular stamp on, and all. My gracious, how queer I feel."

Meanwhile Thomas Hunt solemnly placed the sheet of paper before him, read it through, and turned back to the first line.

"It isn't a hoax," said he. "It's a regular lawyer's letter, and what it tells you is that your old uncle, Samuel Hacker, of London, England, is dead, and you are his heiress to the tune of $100,000. It's down in pounds, but that's the sum in our money."

"Pinch me, Emma Jane!" cried Mrs. Hacker. "I mean it, dear; and if I don't wake up, I'll think it's true."

"Oh, pshaw, ma! It's true enough," cried Emma Jane. "How splendid! When are we to have the money? Oh, isn't it just lovely?"

But Thomas gave a little sigh.

"Mrs. Hacker," he said, "maybe you think a mechanic not rich enough or fine enough for your daughter, now you are so well off as that. If so, say so out and out, and I'll bear it as well as I can."

"Why, Thomas, if I was a queen I'd think you a good son-in-law," said Mrs. Hacker.

"And you, Emma?" said Thomas.

"I shall wait until I get my diamonds on before I take on airs," said his lady love.

Nevertheless the fortune made a change in the programme. It was necessary for Mrs. Hacker to go to England, and Emma Jane must go with her, she said; and, on the whole, it seemed best to postpone the wedding for a while. "It wouldn't be respectful to Uncle Simon to marry immediately," said the mother.

So Thomas had the unhappiness of seeing his lady love leave the shores of her native land, and went back to his shop with a very heavy heart.

However, he worked hard, and many letters comforted him; and at last his Emma Jane returned, gorgeous in the last London fashions; and there was all the bustle of buying a new home, furnishing it, and taking possession of it—and very little time for the lovers to be together.

"You see," said Mrs. Hacker to Thomas Hunt, "Emma Jane is all stirred up. She'll settle down after a while; but young people will be young people, you know."

And poor Thomas did find it out.

"You see, Tom," said Emma, one day, twirling the cheap ring he had given her softly about on her finger. "You see, Tom, somehow, I'd rather not be married for a long while. I don't want you to be angry with me; but I never was a rich girl before—and it's so nice. I get so much attention. I don't want to settle down as an old married woman yet."

"I'll wait, Emma," replied Tom.

"Ah, but—but you see it might be no use," said Emma. "Perhaps I may never want to marry; and if you don't mind taking back that ring, why we can be friends all the same."

"Can we?" said Tom, in a strange tone. "Well, I shall never be your enemy."

And he put the ring into his vest pocket; but he did not trouble the servant to open the door of the big house again.

"What ails Tom, Emma Jane?" asked Mrs. Hacker. "Why don't he come here any more?"

"It's just as well he shouldn't," answered the girl; "and if I could only drop the 'Jane,' ma; I hate it so."

"You didn't use to hate your poor grandma's name," said Mrs. Hacker; "but money has spoiled you, Emma Jane, if ever it spoiled any woman."

"Don't be cross, ma," coaxed Emma. "Tom is very well, but he is common; and you know how elegant young Mr. Vreeland is, and—and he pays me a great deal of attention, ma."

"Oh, that's it," sighed old Mrs. Hacker. "He's cut Thomas out. You've jilted the poor boy."

And now Vreeland came often to see Emma Jane, was her escort everywhere, drove her out, walked with her, sang sentimental songs with his eyes fixed on her face, and all that might he done to show "what his intentions were." And a year from the day on which Mrs. Hacker took possesion of her new house, she was not surprised by hearing that Mr. Vreeland desired to see her alone.

"Yes, I'll go to see him, my dear," said Mrs. Hacker, putting on her best cap at the glass; "but I can't help thinking of poor Tom."

Mr. Vreeland sat in the parlor in exactly the proper attitude, wearing the proper dress, and properly excited—no more. He informed the old lady that he had lost his heart to her daughter, and that, as he believed he had found favor in that young lady's eyes, he desired permission to set the wedding day.

And Mrs. Hacker listened calmly, and answered thus:

"Mr. Vreeland, I think you are what they call a good match for Emma Jane, and I've nothing against you. It shall be as she chooses. Only it is but fair to tell you this: You must take her for herself, for in a week's time we shall leave this house, and I shall go back to my little shop. I've been speculating, and—well, you know how things go sometimes."

"Yes, I know," replied young Vreeland. He turned as pale as death as he spoke, and sat looking down at the carpet.

After a while he said:

"Accept my condolence," and arose and bowed himself out of the front door.

An hour afterward Emma Jane, to whom her mother had told the same story of speculation and loss, received a note, which the Vreeland's black servant had brought to the door. It ran thus:

"MY DARLING EMMA:—You know I adore and must adore you forever; but my habits are extravagant. My father, like your mother, has entered into disastrous speculations, and I will not bind you to a marriage which could result in nothing but misery.

"Yours ever, in despair, REGINALD VREELAND."

Ah, it was all like a dream to Emma. They went back to the old house, and the shop was opened again. The dirty boxes were brushed, the counter oiled, the pins, and buttons, and striped blue elastic, and boxes of cheap thimbles, and the card-board mottoes stamped for working silk, graced the glass case once more.

The same limited number of customers dropped in, and Emma served behind the counter and washed dishes in the back room. She was very, very wretched, and life looked dark indeed to her.

Old Mrs. Hunt and Thomas still lived on the upper floor. The old grandmother told Mrs. Hacker that Tom was beginning to like Fanny Earle, the hair-dresser's pretty daughter.

Sometimes Tom would pass the window, but he never looked toward it.

Emma used to sit behind the counter thinking of him. What a lover she had had, and she had cast him away for a fortune hunter. Her verdict was that she deserved punishment, and she was very sad and meek.

She expected nothing now but to die an old maid, living behind that little shop counter, and never having any admiration or attention again.

In this mood she sat beside her mother one winter evening. The table was spread with the thick stone china ; the brown teapot and the pan of sausage hissed on the stove. The door stood open between the shop and parlor. Notwithstanding all that had happened since, it might have been the same night, a year before, when the letter had come to them which had made such changes, and Emma had even poured out the second cup of tea for her mother, when the door into the hall creaked, and looking up, she saw Tom, big and brown as ever, with such a look in his eyes. But it could not be for her ; she did not deserve it. And Emma dropped her head upon her hands and burst into tears.

Then she felt Tom kneel down beside her and put his arm around her waist.

"Look at me, Emma," he whispered. "Lock at me, my dear. I cannot bear it any more. I never can help loving you, and for all that's come and gone, I believe you do love me a little."

Then Emma found courage to put her hands upon his shoulders and whisper :

"Oh, Tom, I believe I do."

They were married in a very little while, and it was only after the wedding that old Mrs. Hacker, with a very solemn face, informed them that she had a confession to make.

"I haven't lost my money at all, my dears," she said. "I'm half afraid of it, for it seemed to bring unhappiness with it. Yet still it is comfortable to be rich. And now you are married to an honest man, that chose you when you were poor, my dear, we might as well make the most of it, and all go over together—Granny Hunt and all—to the big house the servants are keeping for us, thinking we're off on a journey. I shall never blame myself, and I don't think any of you will blame me, either."

Tom looked at Emma, but she only threw her arms about his neck and hid her face in his bosom and said :

"The money cannot make me any happier than I am, Tom."

And even Grandmother Hunt then declared :

"The house doesn't seem too fine to me now, for there's love in it, and truth in it, and my Tom is as happy as the day."

